# Zoonotic disease risk perceptions and practices related to livestock production and consumption: a qualitative study among animal rearers and veterinary care providers in Northeast India

**DOI:** 10.1136/bmjph-2025-004653

**Published:** 2026-08-12

**Authors:** Melari Shisha Nongrum, Carmenia Khongwir, Arockiasamy Arun Prince Milton, Samir Das, Rajiv Sarkar, Mark L Wilson, Sandra Albert

**Affiliations:** 1 Indian Institute of Public Health Shillong, Meghalaya, India; 2 ICAR RC-NEH, Umiam, Meghalaya, India; 3 ICMR-National Institute for Research in Digital Health, New Delhi, India; 4 University of Michigan, School of Public Health, Ann Arbor, Michigan, USA

**Keywords:** Qualitative Research, Food Safety, Public Health, Zoonoses

## Abstract

**Introduction:**

Zoonotic diseases (ZDs) pose significant global health risks, affecting both public health and the livelihoods of indigenous communities. The Northeast Region of India, with its rich biodiversity, remoteness and porous international borders, is a hotspot for ZDs. Despite extensive human–animal interactions, where risks are likely elevated, there is little literature on zoonoses. This study documented practices related to livestock slaughtering as well as local knowledge and perceptions of zoonoses among livestock rearers and veterinary care providers from such settings.

**Methods:**

We conducted a qualitative study involving 46 participants (livestock rearers, traditional animal healers and veterinary professionals) in Ri Bhoi, East Khasi Hills and Eastern West Khasi Hills districts of Meghalaya state. Livestock rearers with >10 years of experience and veterinary doctors were purposively sampled while traditional healers were identified through snow-ball sampling.

**Results:**

The concept of ‘zoonoses’ was unfamiliar to livestock rearers and traditional healers. Once the idea of zoonotic transmission was introduced, participants acknowledged its possibility and identified mainly meat consumption as a potential route of transmission. Livestock rearers largely practised ‘backyard slaughtering’. They expressed concerns about the lack of preslaughter health checks for livestock, consumption of sick or dead animals and inadequate hygiene in animal sheds as possible risk factors. Livestock rearers and traditional healers possessed local knowledge of common animal diseases and believed that transmission of animal diseases occurs through imported livestock, insect vectors and pig feed. A local practice of relevance was the use of rinse water from meats in households as pig feed.

**Conclusion:**

This study identified awareness and practice gaps regarding ZD risks, underscoring the need for multiprong public health interventions in rural communities. A One Health communication strategy integrating community education, frontline worker training and cross sector collaboration would strengthen disease prevention and enhance early response.

**WHAT IS ALREADY KNOWN ON THIS TOPIC**

- The Northeast Region of India, with its biodiversity and close human–animal interactions, is vulnerable to zoonotic diseases. Over one third of India’s zoonotic outbreaks occur here, yet the knowledge of zoonoses is low and limited to rabies among urban populations.

**WHAT THIS STUDY ADDS**

- In rural Meghalaya, ‘backyard slaughtering’ of livestock is widespread with livestock rearers and traditional animal healers largely unfamiliar with zoonoses. Their perception of zoonotic transmission is limited to consumption of poor-quality meat, with less recognition of other routes of transmission. Livestock rearers’ concerns focus on inadequate health assessment of livestock sold in local markets.

**HOW THIS STUDY MIGHT AFFECT RESEARCH, PRACTICE OR POLICY**

- A One Health strategy, linking animal and human health departments can promote community education and frontline workers’ training, and strengthen prevention and early response during outbreaks. Policy stakeholders could invest in strategies that create basic slaughterhouse facilities at the district or block level from the single central facility that currently exists in the state capital.

## Introduction

 Globally, zoonoses cause 2.5 billion cases of human disease and 2.7 million deaths annually.^1^ Zoonotic diseases (ZDs) pose significant global health concerns by contributing 60% of known and 75% of emerging infectious diseases.^1^ ZD may result from pathogenic microbes that are naturally transmissible from vertebrate animals to humans.^2^ Zoonotic transmission can occur through direct human–animal contact, vectors such as mosquitoes, fleas or ticks, and food and water contamination.^3^ ZDs are classified based on epidemiological features—endemic, epidemic/pandemic-prone and emerging/re-emerging—reflecting their transmission dynamics and public health management needs. They can also be categorised by pathogen types (bacterial, viral, parasitic, fungal) or by the route of transmission.^4,5^ Emerging ZDs are particularly evolving in developing nations, with India emerging as a hotspot due to its increasing population, deforestation, insufficient diagnostic facilities and surveillance, poor practices associated with personal hygiene and improper farming practices.^5^ In India, the ZDs of public health importance include rabies, brucellosis, toxoplasmosis, cysticercosis, echinococcosis, Japanese encephalitis (JE), plague, leptospirosis, scrub typhus, nipah, trypanosomiasis, Kyasanur forest disease and Crimean-Congo haemorrhagic fever.^6,7^ ZDs have a negative impact on human and animal health leading to poorer work performance or economic losses due to the death of animals.^5,8^ Despite this, zoonoses are rarely recognised and inadequately understood.

The Northeast Region (NER) of India constitutes eight states—Arunachal Pradesh, Assam, Manipur, Meghalaya, Mizoram, Nagaland, Sikkim and Tripura—has ~98% of its border with five other countries, and is endemic for many ZDs.^9^ The NER is home to ~40 million people, with over half of the region covered by forests designated as ‘biodiversity hotspots’ within the Indo-Burma and Himalayan hotspots.^10^ Evidence indicates a strong correlation between ‘biodiversity hotspots’ and higher incidence of zoonoses.^11^ Numerous tribal/indigenous communities inhabit this region with their unique sociocultural beliefs and practices, traditional healing systems, meat consumption and traditional livestock rearing systems, resulting in close human–animal contact.^12^ This was demonstrated among bat hunters in the NER who possessed antibodies reactive to two distinct filoviruses—Ebola and Marburg viruses—indicating prior exposure.^13^ Analysis of ZD outbreaks reported under India’s Integrated Disease Surveillance Programme between 2018 and 2023 revealed that more than one-third (36%) of zoonotic outbreaks in India occurred in the NER.^14^ Notably, over one-third of JE cases in India occurred in Assam state alone.^15^ A community-based study assessing seropositivity for scrub typhus among healthy individuals revealed a seropositivity rate of 36.55% in Nagaland, while in Meghalaya, the proportion was higher,^16^ with a case–fatality ratio of 2.3%.^17^ Recently, the first ten cases of leptospirosis—unexpected in hilly regions—were reported from a tertiary hospital in our study area of Meghalaya state.^18^ Furthermore, a study on soil-transmitted helminths (STHs) in Meghalaya using a One Health approach detected human STH species in animals, including 3% prevalence for *Necator americanus* and ~52% *Ascaris* spp. However, as the diagnostic tool could not distinguish between human and pig-associated *Ascaris*, these may represent *Ascaris suum*, with pigs as primary hosts. Zoonotic hookworm *Ancylostoma ceylanicum* (1.6%) was also identified. These findings underscore a complex transmission interface among humans, animals and the environment in high-density NER settings.^19^

While livestock rearers (LRs) are highly susceptible to zoonoses due to their frequent animal contact,^11^ knowledge of zoonoses among LRs in India and other low-and-middle income countries remains low.^20–22^ Existing studies on knowledge of zoonoses in India have primarily focused on the general urban population, with research among LRs largely concentrated in regions outside the NER.^23^ Studies on knowledge of zoonoses in the NER have primarily focused on rabies among urban populations, highlighting a gap in research concerning other pathogens and rural or LRs.^24,25^ To address this gap, this exploratory study employed a qualitative approach, with the aim to examine the livestock slaughtering and the knowledge of zoonoses and modes of transmission among LRs and veterinary care providers in the NER.

## Methods

### Study setting

**Figure 1:**
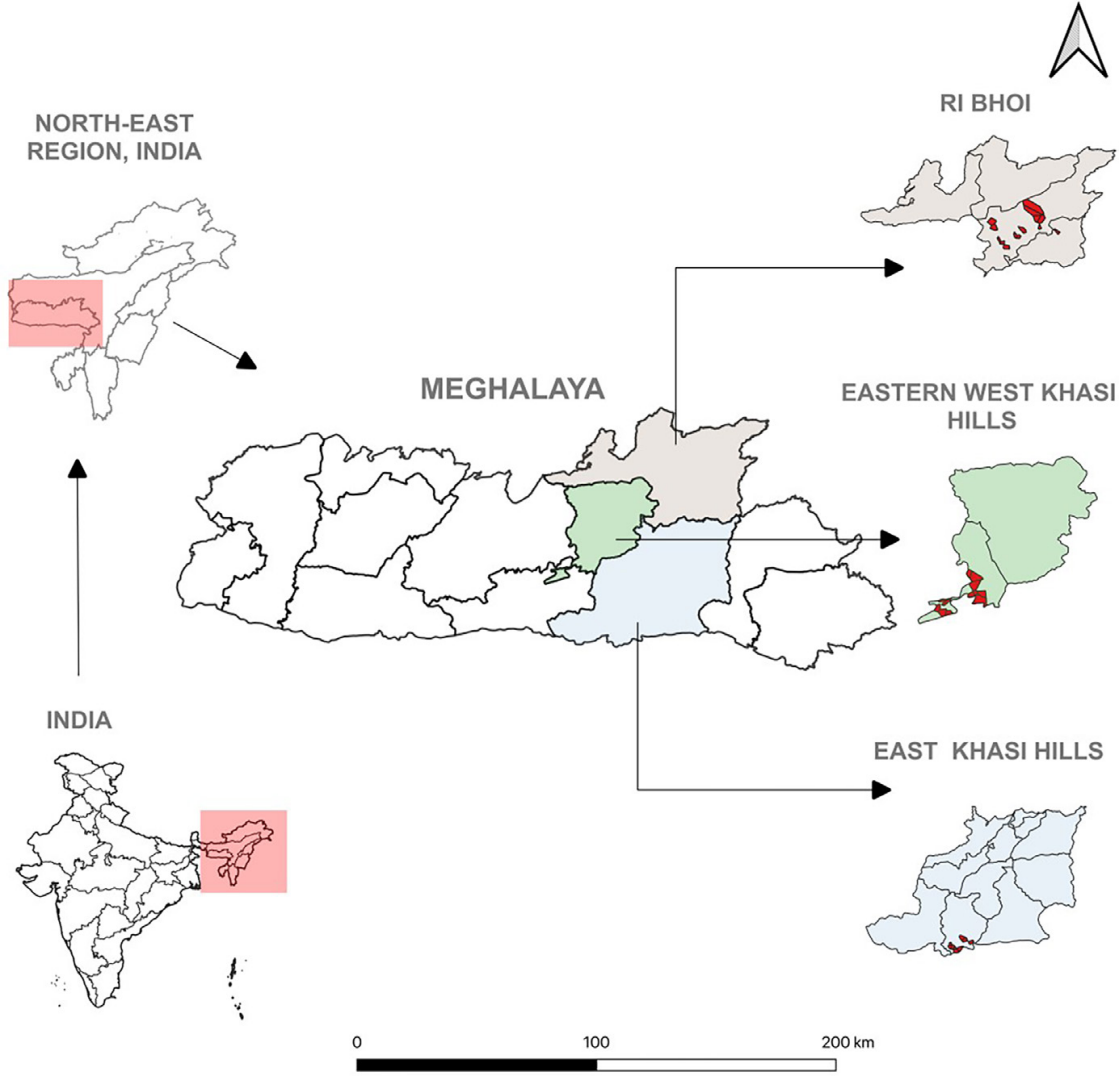
Map of study areas.

This exploratory qualitative study was conducted in Meghalaya, within a larger ongoing One Health project in Meghalaya, one of eight states in the NER, with a population of ~3.5 million people who are predominantly tribal/indigenous people.^9^ Meat consumption occurs in ~80% of the population, along with livestock rearing (mainly cows, pigs, poultry and goats) for food and as subsidiary income in rural areas.^12^ Data collection was carried out in three of the twelve districts in Meghalaya, East Khasi Hills, Ri Bhoi and Eastern West Khasi Hills. Each study district had different topographical features, social cultural practices and cross-border interactions (figure 1).

### Sampling strategy

Nine villages, three from each district, were selected based on proximity to the nearest highway, as road-travel access is a determining factor for availing education and generally influences health-seeking behaviour.^26^ Accordingly, in each district, villages were selected purposively, so that in each district, one village was situated adjacent to the highway, the second was located 5–7 km from the highway and the third village was ≥8 km from the highway. From each village, three to four LRs, >10 years of experience who reared domestic animals (poultry, cattle, pigs) were purposively selected for the study. A field volunteer from each community aided in the selection of these participants.

While the initial plan was to include only LRs, veterinary care providers such as animal traditional healers (THs) and veterinary professionals (Vets) were added after a few initial LR interviews. This decision stemmed from the LRs’ acknowledgement of the role that THs and Vets play in caring for and treating animal diseases. A snowball sampling method was used to identify the THs who treated animals, as not all THs treated animals. To obtain perspectives of Vets on zoonoses, in-depth interviews (IDIs) with veterinarians posted by the Government of Meghalaya in the study areas were conducted as veterinary services in rural areas are largely provided by the government.

### Data sources and collection methods

A topic guide was developed and adapted for IDIs with LRs, THs and Vets. The topic guide probed on the perception and knowledge regarding ZD transmission, practices and potential factors leading to animal–human pathogen transmission. It was iteratively adapted after regular team meetings to understand the key emerging themes. The interviews were conducted between April 2023 and June 2024, at each participant’s home, village community hall or office of the village council. Each interview lasted for 35–40 min. All participants were interviewed individually, except in one village where a focus group discussion with LRs was conducted.

The IDI data were collected by two female tribal researchers who had postgraduate qualifications in social work and were from the same ethnic communities as the participants. Their cultural familiarity helped build rapport with participants. These researchers had prior experience in collecting qualitative data, but were trained on interviewing skills, transcription and qualitative data analysis, as well as research ethics. Both researchers were fluent in Khasi (local language spoken by majority of people in the study areas), in addition to English. Other members of the team also spoke other languages, such as Garo, which aided data collection in the villages of East Khasi Hills district, where a sizeable proportion of residents were Garo-speaking. The local names of animal diseases were translated into English and verified by veterinarians fluent in Khasi and Garo for the analyses.

### Data analysis

The entire research process was conducted following the Consolidated Criteria for Reporting Qualitative Research (COREQ) guidelines.^27^ Data were analysed using the thematic analysis approach. The oral recordings were first transcribed in the local languages and then translated to English. To ensure data reliability, another member of the research team, who did not transcribe the data, checked the quality of transcription. An inductive coding process was employed to code the data using Taguette V.1.4.1,^28^ an open-source software for qualitative data analysis. To ensure validity, all codes were verified by a senior researcher who re-examined the codes vis-à-vis the transcripts before they were further analysed into categories and themes. Emerging categories and themes were developed after thorough discussion within the research team.

A written informed consent was obtained from all respondents prior to their participation for interviewing and audio recording. The participant information sheet and informed consent forms were available in the local languages (Khasi/Garo) as well as in English.

To anonymise the participants, their names were replaced with a common acronym based on the occupation such as ‘LR’ for livestock rearer, ‘TH’ for traditional healer and ‘Vet’ for veterinarian.

## Results

**Figure 2:**
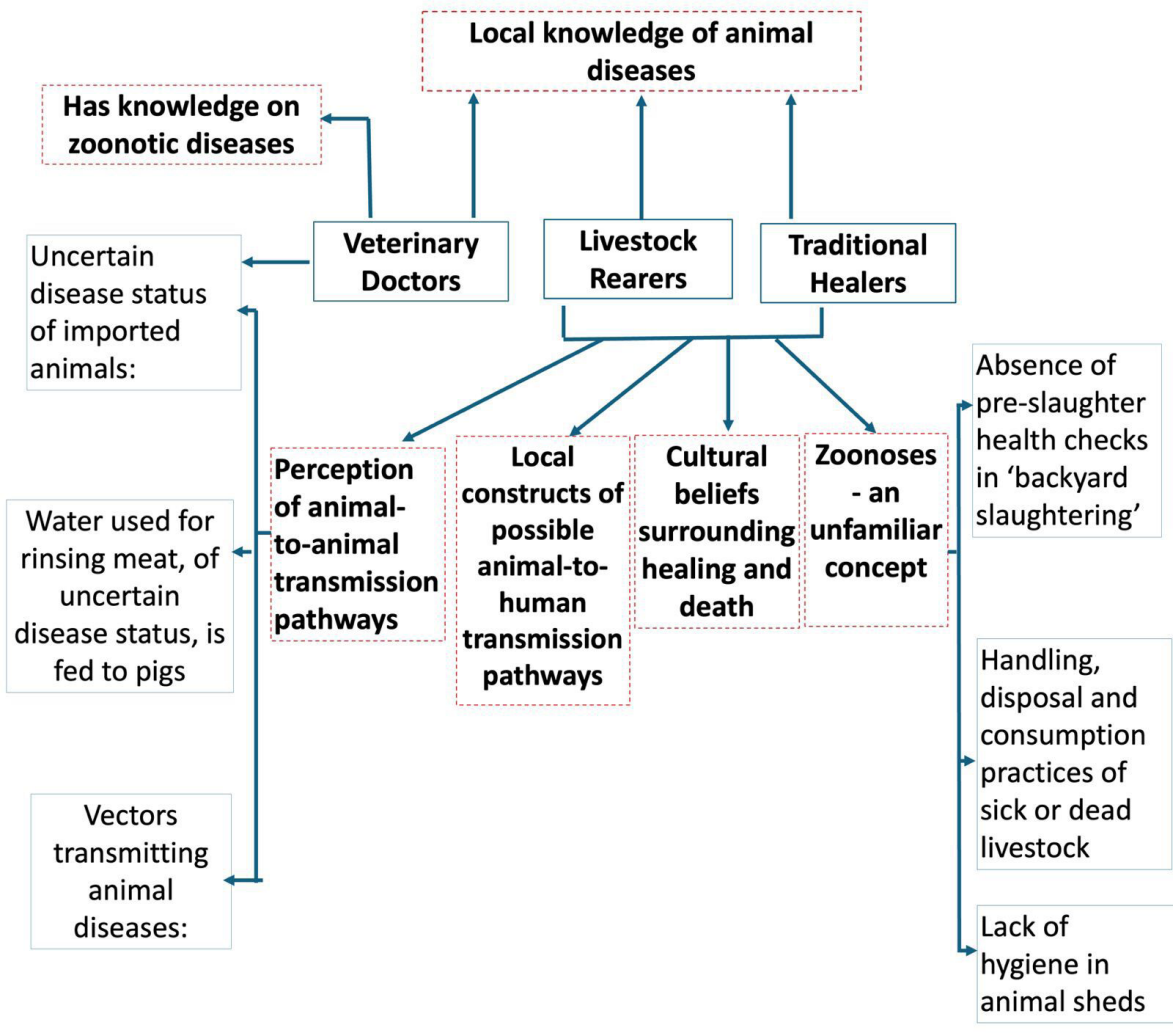
Schematic diagram representing perceptions of zoonoses.

The study participants consisted of 36 LRs (16 females, 20 males), who owned different types of livestock such as cattle, pigs, poultry and ducks. There were also seven THs (one female, six males), who treated cattle, pigs and poultry, and three Vets (one female, two males). Results are presented under four main subheadings: (1) local knowledge of animal diseases and perception of animal-to-animal transmission pathways; (2) cultural beliefs surrounding healing and death; (3) knowledge of zoonoses; and (4) local constructs of possible animal-to-human transmission pathways. Insights that overlap among participants are highlighted in figure 2. The figure provides a schematic representation of participants’ perceptions related to ZDs.

### Local knowledge of animal diseases and perception of animal-to-animal transmission pathways

LRs were well-versed in identifying common diseases among their animals (eg, black quarter (*Clostridium chauvoei*), foot-and-mouth disease (*Alphavirus*), lumpy skin disease (LSD) (*Capripoxvirus*) and cysticercosis (‘measly beef’ in cattle and ‘measly pork’ in pigs), referring to them by their local names. They could name other symptoms such as diarrhoea, fever, cough, wounds and skin rashes among the livestock. Through the signs and symptoms observed in these animals, LRs were sometimes able to identify the disease condition and differentiate one illness from another. *“*Another illness which we know is ‘jingpang ïong’ [black quarter], a disease seen in cows. The signs and symptoms are bloating and the animal’s head would get swollen, and it is known as ‘jingpang ïong’ [black quarter]”. Veterinarians also corroborated that LRs could recognise signs and symptoms of common livestock diseases. “We can say that LRs are aware, especially farmers who are in touch with us all the time. They come to us to inform us that this and that has happened so they do have knowledge about common livestock diseases*.”* For livestock health issues, LRs first relied on home remedies; if conditions worsened, those in remote areas turned to THs, while those in accessible villages, they visited the local animal husbandry dispensary.

### Uncertain disease status of imported animals

Movement of livestock increases the risk of ZDs.^29^ THs through their experiential acumen, shared that poultry imported from other states may harbour infections, not immediately visible. One TH observed, “Poultry imported from outside the state may be infected with diseases and affect our local poultry when they are kept in the same coop*.”*, They also implied that pigs sourced from outside Meghalaya could be infected. LRs and Vets shared similar concerns, emphasising that imported livestock could introduce diseases if not properly screened at the point of entry. A Vet cited an example, the LSD outbreak in Meghalaya in 2023 was ‘likely triggered by importation of infected cattle’.

### Vectors transmitting animal diseases

Vector borne diseases such as Trypanosomiasis, LSD, bluetongue or epizootic haemorrhagic disease are transmitted by insect vectors such as tabanid flies, *Culicoides* midges.^30^ Although THs did name specific diseases, they reported the presence of insects ‘like mosquitoes’, prevalent in monsoon that bite cattle and pigs. These insects resemble ‘mosquitoes, but are larger, with a highly toxic bite’ that spreads throughout the animal’s body, often leading to its death. One TH stated, *“*These insects transmit the disease by sucking blood from one animal and then moving on to another, thereby spreading the infection*”*.

### Water used for rinsing meat, of uncertain disease status, is fed to pigs

Any pathogen present in the water used during slaughter and preparation of muscle/meat/offal poses pathogen transmission risk.^31^ Tribal communities of Meghalaya avoid waste and consume all parts of a slaughtered animal. For example, pigs are eaten from nose to tail, with blood used to make sausages or cooked with rice as a local delicacy. LRs reported that rinse water from washing meat is traditionally collected with other food remnants and fed to pigs. They expressed concern that if meat from a sick animal were unknowingly purchased and its rinse water fed to pigs, infection could spread. One LR said, “while cleaning this meat, the water which is collected is fed to our pigs, so this is a possible reason for transmission of diseases from animal to animal*.”* Some LRs also stated that during pig disease outbreaks, they refrain from feeding rinse water from meat to the pigs, discarding it as a precautionary measure.

### Cultural beliefs surrounding healing and death

Certain older LRs and THs believed that diseases from humans could be transmitted to animals. They explained this phenomenon as—when a livestock dies in a household of a sick person, it is believed that the disease has passed from the sick person to the livestock due to divine intervention. Following the death of the livestock, the sick person recovers. When this happens, it is accepted positively, interpreted as a ‘lucky’ alternative by the household and community, but is not recognised as a possible animal–human transmission pathway. A TH stated what he believed, “Our ancestors have instilled a belief in us that it is better for an animal to die than its owner. According to this belief, if someone in a household is seriously ill, and an animal dies instead of the person who has fallen ill, then this signifies that the owner will recover. It is believed that the animal has absorbed the illness from its owner, thereby curing him. This belief is deeply ingrained in our culture*.*” This belief was strengthened each time such events were observed in their communities.

### Zoonoses: an unfamiliar concept

In the Khasi and Garo communities, no word or phrase exists that describes zoonoses as a single idea or concept in their local language. Therefore, a very basic explanation had to be made about the meaning of zoonosis. The researchers explained that there are certain animal diseases that could be transmitted to humans from infected animals and cause illness through handling infected animals, consumption of improperly cooked meat, drinking unboiled milk and consuming infected meat. Without mentioning any specific diseases, it was conveyed that scientists used the term ‘zoonotic diseases’ to refer to such diseases. Generally, the LRs did not have specific knowledge about pathogen transmission from animals to humans, and the concept of zoonosis was unfamiliar to them. As expressed by an LR, “I don’t really know of diseases which can be transmitted from animals to humans”. While LRs generally lacked specific knowledge on zoonosis, on probing and explaining the meaning of zoonosis by taking examples from other parts of the world, both LRs and THs acknowledged its possibility. However, they did not perceive themselves as being more susceptible to such infections, despite being in close contact with livestock animals. “I heard it from my friends who are also rearing livestock. They said that only pigs can transmit the disease to humans. I have never seen this but I have only heard from other people.”

Subsequently, after the researchers provided a brief idea on the concept of zoonoses as indicated, a few LRs mentioned that they observed occasions when animals and their handlers were ill at the same time. One LR mentioned, “when humans get fever, it is during the exact same time that there is a disease outbreak in the chickens and pigs. At present also, in the village many of the children are getting fever. There is an outbreak going on, where the chickens and pigs are showing symptoms of cough and fever and these animals eventually die from it.” However, it remains uncertain to the LRs whether these conditions represent true zoonoses according to the definitions and understanding of biomedical science.

THs often handled sick livestock animals with bare hands, unaware of health risks. Vets reported widespread lack of understanding of ZDs and their transmission pathways to LRs and rural populations, despite frequent human–animal contact. Vets stated that awareness programmes typically focus on common livestock diseases. JE and rabies are diseases that they have observed and reported. “I explain to them about zoonotic diseases and most of them are not aware that it can spread from animals to humans which makes them at risk for these diseases. Besides, the increase in population and to meet economic needs, there is a need to rear animals but information about zoonotic diseases is very low.”

### Local constructs of possible animal-to-human transmission pathways

After the research team provided a brief explanation on the concept of zoonoses that there are certain animal diseases that could be transmitted to humans from infected animals and cause illness through handling infected animals, drinking unboiled milk or consumption of improperly cooked meat/produce, the LRs and THs reflected on this and offered their perspectives on the possible transmission pathways, primarily via meat consumption. These perceived risks were categorised into three main themes: (1) absence of preslaughter health checks in ‘backyard slaughtering’; (2) handling, disposal and consumption practices of sick or dead livestock; and (3) lack of hygiene in animal sheds.

### Absence of preslaughter health checks in ‘backyard slaughtering’

The most common livestock slaughter method in rural areas of Meghalaya is ‘backyard slaughtering’ where animals are slaughtered using traditional methods in open or semisheltered ‘spaces’, with limited infrastructure. This practice is widespread, as there is only one official government slaughterhouse located in the state capital. The absence of preslaughter health checks of livestock in ‘backyard slaughtering’ increases public health risks.^31^ LRs and THs commonly perceived zoonotic transmission through meat bought from local markets, mostly slaughtered in ‘backyard slaughter spaces’. These unregulated livestock slaughter houses, located in villages or near local markets, operate without any veterinary inspection. An LR stated, “Maybe this transmission is because we buy meat from the market place and, who knows, maybe the meat that we bought is from a sick and infected animal. There is no one to check”. Vendors may inadvertently or knowingly sell meat from diseased animals, posing risks of zoonotic infection, a concern raised by both LRs and THs.

Furthermore, LRs reported that raw meat is sold openly without any quality or food safety measures, “…so, we buy this meat and we consume it, this can be one of the reasons for transmission of diseases.” The THs also echoed these concerns, specifically questioning meat quality, slaughtering methods, absence of professional veterinary checks prior to slaughter and potential for sale of infected meat in markets.

### Handling, disposal and consumption practices of sick or dead livestock

LRs and THs generally believe that dead animals can transmit diseases; hence, some LRs do not touch the carcass. One LR said: “We never touch the dead animals with our bare hands. And when they die, we would just tie the body of the dead animal with a rope and pull it to the place of burial”. LRs reported burying deceased livestock to prevent disease spread, though villages lack designated burial sites. Some animals are buried in distant community forests, while landowners use their own plots. Typically, a six-foot hole is dug for the carcass. One LR noted, “if we don’t bury them properly, the dogs will eat the dead chicken.” In one village, a misconception persists that burying animal in gardens with squash or banana plants prevents disease, as the plants absorb the decayed matter: “We bury them in a separate plot of land, which we have. We dig a deep hole and bury them. On top of this we plant a banana tree, so that the disease does not spread.”

Some participants believed that consuming deceased livestock could transmit diseases, and therefore refrained, with one stating, “when we already know that they die from a disease then how can we eat them, right?” A few LRs shyly acknowledged hearing that in other communities, people sometimes consume the meat of livestock that died or showed signs of illness. Participants agreed that while some people do eat such meat, it is largely uncommon among Khasi or Garo communities. As one LR shared, “There are places I know that once an animal has symptoms of an illness, they would slaughter the animal and consume the meat.” In such instances, only meat or body parts that appear safe from visual inspection, that is, meat or body parts not ‘discoloured’, were consumed. Among LRs, discussion of effects was limited, though one elder who recalled a past incidence of stomach illness after eating meat of deceased animals.

Few THs have observed that people in some communities consume meat of sick and deceased livestock with varying effects. One TH noted no immediate harm, another said that there could be long-term health impacts. “Some individuals still consume meat from animals they know are sick, and while it may not have immediate effects, after 10 or 12 years, it could potentially lead to diseases in people, which we are not aware.”

Veterinarians also reported hearing of people consuming meat of deceased livestock, without knowing the animal’s cause of death. “In the past few days, I have heard that in one of the villages there was a truck (vehicle) carrying cows and one of the cows died; the people of the village feasted on the meat of the dead animal. I don’t know whether the animal was infected or only died from an injury”.

### Lack of hygiene in animal sheds

LRs perceived poor hygiene in animal sheds as a source of infection for both animals and humans. One LR in East Khasi Hills district, where cattle sheds are often part of the house, stated: “If we do not keep our surroundings clean and if we do not maintain proper hygiene in the place where we keep our animal, then there is a possibility of disease transmission to humans.” Researchers observed that animal sheds were cleaned with water but lacked drainage for faecal waste. LRs also noted that unhygienic surroundings attract flies and mosquitoes, could spread diseases. While several mentioned vector risks, only one LR specifically linked JE to mosquitoes transmitting infection from pigs to humans.

## Discussion

This study revealed that LRs and THs in rural Meghalaya were unfamiliar with the concept ‘zoonosis’. When probed, some acknowledged the possibility of pathogen transmission from animals to humans, particularly through meat handling and consumption of infected meat. Participants showed little or no recognition of other important zoonotic transmission routes, such as via direct human–animal contact, blood-sucking arthropod vectors or humans drinking infected water.^6^ These findings differed from other studies in south India which indicated that LRs in south India had better knowledge of zoonoses and good husbandry practices towards ZDs prevention and control.^23^ In Nepal, smallholder farmers showed awareness of zoonoses but failed to adopt safe practices, reflecting a disconnect between knowledge and practices.^32^ Among comparable sub-Saharan Africa livestock rearing populations, the LRs had better knowledge of zoonoses as compared with our study. Also, while there was some similarity in understanding that consumption of untreated livestock products (ie, milk, meat or eggs) was perceived as route of transmission, perceptions also differed, as in sub-Saharan Africa, they perceived that shared living accommodation with animals and attending to parturition were perceived transmission routes.^33^ Limited knowledge of zoonosis in Meghalaya may stem from inadequate ZD education initiatives among LRs. Current information education and communication materials from the Meghalaya Animal Husbandry and Veterinary Department emphasise livestock care and prevention, but little on zoonoses.^34^ Vets indicated that community sensitisation around zoonoses remains limited, leading LRs and THs to often neglect precautions while handling animals. Frequent human–animal interactions coupled with limited understanding of transmission risk highlight the need to strengthen zoonoses knowledge, especially in rural communities. Engaging local stakeholders, such as ‘Dorbar’ (village council)^35^ and village health councils,^36^ is crucial. *Dorbar* successfully supported the COVID-19 response in Meghalaya^37^ and similar community engagement aided rabies elimination in the NER State of Sikkim.^38^ In addition, THs can help disseminate information on ZD prevention. As trusted community members, people prefer to consult them and value their advice. Collaborating with THs offers a valuable opportunity for effective community-level awareness and intervention.

Backyard slaughtering remains common in Meghalaya due to limited abattoirs.^39^ The absence of preslaughter health check-ups by trained personnel and inadequate sanitation in village slaughter houses concern both LRs and THs, as they heighten risks of pathogen transmission and meat contamination. While infrastructural challenges hinder the establishment of abattoirs across rural Meghalaya, community-based collectives of slaughterhouse workers and meat handlers could adopt safe and sustainable practices within existing backyard slaughtering spaces.^40^ Government funding exists to modernise traditional slaughter houses^41^ and aligning these with health guidelines would strengthen slaughtering and meat handling systems, reducing transmission risk of ZDs.

Consumption of sick or dead livestock was also identified as a possible source of zoonotic transmission. Similar practices and perception of zoonoses and associated risks were found in Kenya^42^. Although consumption of potentially infected meat presents a ZD risk, proper cooking could provide protection.^42^ In Meghalaya, meat is typically well-cooked, with little consumption of ‘rare’ meats. Good practices such as avoiding sick or dead animals should be reinforced alongside sensitisation about zoonotic risks from consuming unhealthy livestock. During outbreaks, bans on livestock sales are common, but these cause heavy losses for rearers and retailers. Preparatory efforts are therefore needed to help communities understand the rationale behind bans or culling, without which they risk greater transmission and under-reporting.

LRs expressed concerns about zoonosis risks from poor hygiene in animal sheds, often lacking proper drainage and waste disposal. Evidence shows improved water, sanitation and hygiene around sheds and households reduces ZDs.^43^ Current programmes in Meghalaya—Jal Jeevan Mission (piped water)^44^ and Swachh Bharat Abhiyan (sanitation) do not address water use by animals.^45^ The National One Health Mission emphasises integrated health outcomes across humans, animals and environments, making it vital to engage LRs in recognising these interconnections. Recognising animals as water users, polluters and pathogen reservoirs^46^ alongside promoting protective equipment like specialised gloves for meat handlers is essential.^47^ Infrastructure improvements in drains and waste disposal can be linked to the Mahatma Gandhi National Rural Employment Guarantee Scheme, enabling community driven assets tailored to local needs. Similar linkages to animal husbandry have been initiated in other states^48^ and could also be implemented in Meghalaya.

Importation of livestock is profitable but poses public health risks. LRs noted that local livestock could be infected when imported livestock are housed together, especially without proper veterinary inspections, leading to economic and public health impacts.^8^ Veterinary inspections of animals transported across states are mandatory in India, as they may harbour pathogens even without symptoms.^49,50^ Meghalaya’s borders with Assam and Bangladesh allow frequent livestock movement facilitating ZD transmission.^50^ Although livestock transportation requires veterinary health and transit certificates, under the guidelines of the Transport of Animal, Rules, 1978,^49^ yet enforcement in remote rural areas is weak, and illegal cross-border sales have been reported.^51^ Engaging with LRs and communities to enhance community awareness of ZDs and the risks of zoonoses of cross-border trade can support to curb introduction and emergence of new pathogens.

Among limitations of the present study, the knowledge of and perspectives on zoonoses of meat handlers was not specifically evaluated, even though they are ‘middlemen’ who purchase live animals from the LRs and slaughtered meat from butchers. In addition, we only studied information gathered from three of the twelve districts in the state, which could limit the generalisability of our findings. Nevertheless, the insights gained provide useful understanding of knowledge and practices that are likely similar throughout the region.

In conclusion, this study highlights that zoonoses remain an unfamiliar concept of minimal concern, especially among LRs and THs. These people perceived that zoonotic transmission is primarily limited to certain consumption of certain meat products. Our findings suggest the need for a multipronged approach to ZD control through coordinated, cross-sectoral efforts that link the public health, veterinary, wildlife and environmental sectors. Strengthened surveillance, community engagement, vaccination, environmental management and multidisciplinary research addressed simultaneously can enhance the prevention and control measures and reduce zoonotic impacts, particularly in high-risk populations. A One Health communication strategy integrating community education, frontline worker training and cross-sector collaboration would strengthen prevention and enable early response. Policy stakeholders should also ensure basic slaughterhouse facilities at district or block levels, linked to the central facility in the state capital.

## Data Availability

Data are available upon reasonable request.
